# Coccidioidomycosis in Biopsies with Presumptive Diagnosis of Malignancy in Dogs: Report of Three Cases and Comparative Discussion of Published Reports

**DOI:** 10.1007/s11046-015-9948-4

**Published:** 2015-09-29

**Authors:** Rafael Ramírez-Romero, Rolando Antonio Silva-Pérez, Jorge Lara-Arias, Cecilia Ramírez-Hernández, Iván Alberto Marino-Martínez, Álvaro Barbosa-Quintana, Alfonso López-Mayagoitia

**Affiliations:** Posgrado Conjunto Agronomía-Veterinaria, Facultad de Medicina Veterinaria y Zootecnia, Campus de Ciencias Agropecuarias, Universidad Autónoma de Nuevo León, Av. Francisco Villa s/n, Ex-Hacienda el Canadá, C.P. 66050 Gral. Escobedo, N.L. México; Banco de Hueso y Tejidos, Hospital Universitario “Dr. José E. González”, Universidad Autónoma de Nuevo León, Monterrey, N.L. México; Centro de Investigación y Desarrollo en Ciencias de la Salud (CIDICS), Universidad Autónoma de Nuevo León, Monterrey, N.L. México; Departamento de Anatomía Patológica, Hospital Universitario, Facultad de Medicina, Universidad Autónoma de Nuevo León, Monterrey, N.L. México; Atlantic Veterinary College, University of Prince Edward Island, Charlottetown, PE Canada

**Keywords:** Coccidioidomycosis, Dog, Neoplasia, Surgical pathology, Mexico

## Abstract

Coccidioidomycosis is a respiratory fungal infection with occasional systemic dissemination. The disseminated coccidioidomycosis is considered a multifaceted disease. In medicine, disseminated coccidioidomycosis is included within a group of infectious diseases that have been referred as the great imitators. In many cases, malignancies are included in the presumptive diagnosis. In veterinary medicine, disseminated coccidioidomycosis is common in dogs. Nonetheless, despite of being a diagnostic dilemma, disseminated coccidioidomycosis is underestimated and frequently not included into differentials, even in endemic zones. Herein, we describe three cases of granulomatous inflammation caused by *Coccidioides* spp. which were masquerading malignancies in dogs (0.39 %). The presumptive diagnoses in these cases were osteosarcoma, lymphoma and neurofibroma, respectively. A PCR assay employing tissues in paraffin blocks resulted positive for *C*. *posadasii* in one of these cases. A comparative discussion on the ambiguous clinic-pathological presentation of disseminated coccidioidomycosis in dogs and humans is included.

## Introduction

Neoplasia is of major relevance in both medicine and veterinary medicine. For diagnosis, even nowadays when molecular procedures are commonly employed, surgical pathology is irreplaceable [1]. Slide interpretation by a pathologist is still the most useful and precise procedure for malignancies diagnosis [1]. Clinicians require the pathologist to offer a prognosis in a neoplasm [1]. Furthermore, histopathology can also be the guidance if the presumption was equivocal, such as in neoplasm-like cases of coccidioidomycosis [2]. Indeed, histopathology is a standard gold test for coccidioidomycosis [2, 3]. Identification by molecular procedures is not available routinely for coccidioidomycosis [2, 4]. Nowadays, there are few reference laboratories employing PCR to clinical samples [2, 4, 5]. Fortunately, for diagnosis, histopathology is still superior to molecular techniques such as PCR [2, 4, 5]. In fact when tissue is available, histopathology is the first procedure routinely employed to diagnose all systemic fungal infections [2–4].

Coccidioidomycosis is a respiratory and systemic mycotic disease, highly relevant in public health [6–8]. The specialized requirements of *Coccidioides* spp. confine the fungus to limited zones of high endemicity. These zones are characterized by arid alkaline lands with limited rainfalls, high summer temperatures and few freezing days in winter. Soils with these characteristics prevail in the southern USA, particularly in the “lower Sonoran life zone” [6, 9]. The states of Arizona and California are considered high endemic areas. However, New Mexico and Texas are also emerging [6, 7]. In Mexico, coccidioidomycosis is most prevalent in the states neighboring USA [3, 8, 9]. Infections with *Coccidioides immitis* are predominant in the Northwest, whereas in Northeast *Coccidioides posadasii* is more prevalent [10]. The state of Nuevo León in the Northeast, bordering with Texas, USA, is the state with the highest incidence of coccidioidomycosis in humans in Mexico [8]. All of the cases herein included belong to dogs from the city of Monterrey, principal city of Nuevo Leon, Mexico.

We report here three cases of dogs clinically suspected of having neoplasia; however, surgical biopsies confirmed that the problem was unsuspected granulomatous inflammation caused by disseminated coccidioidomycosis.

The material herein included corresponds to 765 dog biopsies with presumptive diagnosis of neoplasm between April 1, 2010, and March 31, 2015. Samples were submitted by veterinarians with private practice in small animals in Monterrey, Mexico. Tissues were submitted in 10 % buffered formalin. Histology procedures were conventional with routine H&E stain and in the cases herein presented also periodic acid Schiff (PAS) and Gomori methenamine silver (GMS) stains were employed.

Among the 765 biopsies with presumptive diagnoses of neoplasm, three cases of coccidioidomycosis were recognized (3/765 × 100 = 0.39 %). The 762 cases of neoplasia confirmed by histopathology (762/765 × 100 = 99.60 %) reveal a higher correspondence between biopsies with presumption of malignancy and histopathological confirmation. Therefore, coccidioidomycosis is reasonably unexpected in biopsies with clinical presumption of neoplasia. These three coccidioidomycosis cases are herein described.

### Case 1

An 18-month-old male German shepherd was presented to the veterinarian for progressive weight loss and weakness that eventually lead to prostration. During clinical examination, the veterinarian noticed that the masseter muscles were atrophied and several painless nodular lesions (0.5–1 cm) involving the tarsal, phalanges and lumbar regions were evident. Radiographs of affected bones revealed low-density proliferative osseous changes which were interpreted as consistent with neoplasm. The presumptive diagnosis was osteosarcoma. Six small (0.3–0.5 cm) tissue samples were taken from affected areas, fixed in 10 % buffered formalin and submitted to histopathological studies. Microscopically, all biopsies showed extensive connective tissue proliferation heavily infiltrated with macrophages, giant multinucleated cells and some neutrophils and lymphocytes. Most remarkable was the presence of numerous conspicuous PAS- and GMS-positive oval spherules (10–40 µm diameter) with thick refractile walls containing small bodies (endospores). Most of these spherules were intact, but few of them were broken releasing the endospores in the surroundings. These spherules were interpreted as fungal organisms with morphologic features of *Coccidioides* spp. Morphologic diagnosis was severe granulomatous periostitis and deep dermatitis, chronic, locally extensive with numerous intralesional fungal organisms consistent with *Coccidioides* spp.

### Case 2

**Fig. 1:**
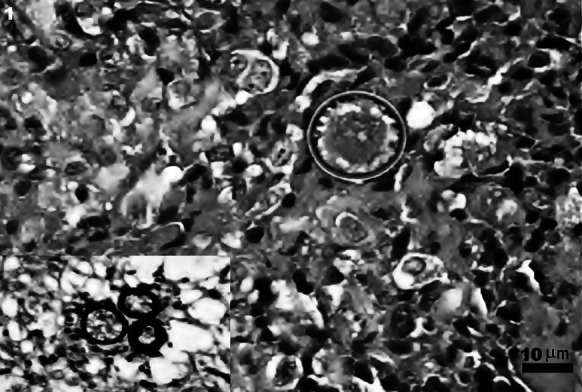
Case 3, dog with suspicion of neurofibroma. There is one spherule with thick and refractile cell wall. The endospores contained within are ill defined. The inflammatory reaction is composed by epithelioid macrophages and lymphocytes; the proliferation of fibrous connective tissue is prominent. H&E *bar* 10 µm. The *inset* depicts the special stain with three organisms in a pyogranulomatous reaction in case 2. GMS. The image is representative of all of the cases here included

A 12-month-old female Boxer was presented to the veterinarian for a progressive swelling of the left hind leg. On physical examination, a movable non-painful mass was recognized in the popliteal region. No other changes were noticed during the physical examination. The veterinarian interpreted the growth on the hind limb as an enlarged popliteal lymph node. The whole lymph node was surgically excised. On palpation, the node had a soft texture with some hemorrhages on cut surface. The presumptive diagnosis was lymphoma. The lymph node was fixed in 10 % buffered formalin and submitted for histopathological examination. Microscopically, the specimen was a lymph node in which the histological architecture was notably effaced by fibrosis and an intense infiltration of macrophages, giant multinucleated cells, lymphocytes, plasma cells and neutrophils. The inflammatory response was more evident at the hilum of the node where fibroplasia was also marked. Embedded in this granulomatous reaction there were numerous PAS-/GMS-positive spherules ranging in size from 10 to 40 µm in diameter. These fungal structures had thick birefringent walls containing round endospores (Fig. 1, inset). Morphologic diagnosis was severe granulomatous/pyogranulomatous lymphadenitis, chronic, multifocal to coalescent with numerous intralesional fungal organisms consistent with *Coccidioides* spp.

### Case 3

A mature (age unknown), female Labrador retriever with a several months history of progressive weakness, ataxia and prostration was presented to the veterinary clinic. On physical examination, the dog appeared in poor condition. Moreover, a subcutaneous non-ulcerated firm mass (7.0 × 6.0 cm) was noted on the left side thorax (ribs 9–11). This mass was non-painful and non-movable. Physical exam also revealed weakness and hypoesthesia affecting the left hind leg. Radiographic study exposed locally extensive osteolysis of affected ribs. The mass was partially removed and submitted for histopathological examination. The presumptive diagnosis was neurofibroma. Microscopically, the specimen was composed by skin and subcutaneous tissue severe and diffusely infiltrated with macrophages, lymphocytes, plasma cells, giant multinucleated cells and few neutrophils. These inflammatory infiltrates were forming well-delineated granulomas with necrotic centers surrounded by phagocytic cells and encircled with a tick band of connective fibrous tissue. These granulomatous lesions also contained numerous PAS-/GMS-positive fungal structures morphologically consistent with *Coccidioides* spp. (Fig. 1). Morphologic diagnosis was severe granulomatous deep dermatitis, fasciitis and periostitis (ribs 9–11), locally extensive, with numerous intralesional fungal structures compatible with *Coccidioides* spp.

### Molecular Procedures

**Fig. 2:**
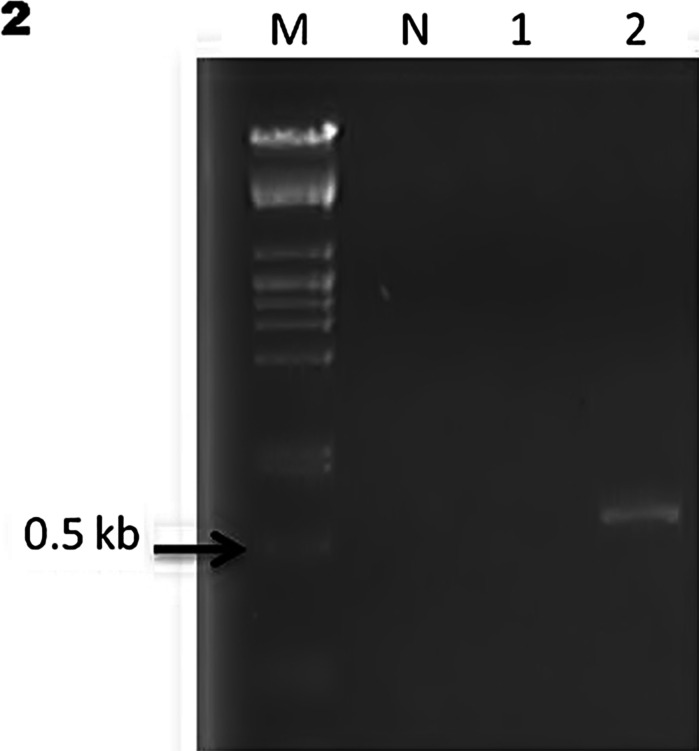
PCR amplification from two paraffin-embedded tissues. *Lane M*, DNA molecular weight marker, *lane N*, negative control. *Lane 1*, sample case 2 and *lane 2*, sample case 3 with an amplicon of 634 pb. The result corresponds to *C*. *posadasii*

For PCR, total DNA was extracted from the paraffin-embedded tissues using the ReliaPrep™ FFPE gDNA Miniprep System (Promega Corp.). Paraffin blocks with more presence of *Coccidioides* spp. were selected. The primer set was Coi9-1F (5′-TACGGTGTAATCCCG ATACA-3′) and Coi9-1R (5′-GGTCTGAATGATCTGACGCA-3′) as previously reported [11]. PCR conditions were as follows: 1 cycle at 94 °C for 3″ followed by 35 cycles at 94 °C for 30′, at 60 °C for 30′ and at 72 °C for 45′ with a final step at 72 °C for 3″ [11]. Unfortunately, tissues were available only from cases 2 and 3; the blocks included were those with major evidence of *Coccidioides* spp. in tissues (cases 2 and 3). The amplicons reported for the selected primers are 720 bp for *C*. *immitis* and 634 bp for *C*. *posadasii* [11–13]. Due to scarcity of tissue in paraffin blocks, the isolated DNA concentration was not enough to amplify the sample 1 (case 2). However, it was possible to amplify the second sample (case 3) with an approximately 634-bp amplicon, corresponding to *C*. *posadasii* according to previous reports [11–13] (Fig. 2).

## Discussion

Coccidioidomycosis is a renowned clinical impersonator of malignancy in human and veterinary medicine [5, 14–17]. A comparative list of organs affected with coccidioidomycosis masquerading malignancy in human and dogs is summarized in Table 1. When veterinary practitioners are confronted with the classic algorithm of neoplasm/inflammation, histopathology in a biopsy is by far the most accurate method [2]. Furthermore, histopathology is the procedure of choice for systemic fungal infections [2, 4].

**Table 1:** Comparative cases of coccidioidomycosis in humans and dogs with presumptive diagnosis of neoplasia

Human beings Tissues, organs	References	Dogs Tissues, organs	References
Lymphoid	Aviles-Salas et al. [18]; suspected lymphoma; Cannella and Vinetz [19]; suspected lymphoma	Lymphoid	Jeroski [20]; coccidioidomycosis concomitant with disseminated lymphoma [*] Suspected lymphoma case 2 herein included
Bone	Arora et al. [21]; multiple myeloma or bone metastasis; Caraway et al. [22]; primary bone neoplasm Li et al. [23]; suspected primary bone neoplasm Smitherman and Ritter [24]; presumptive pelvic sarcoma Huang et al. [25]; presumptive metacarpal enchondroma	Bone	Shubitz and Dial [16]; suspected bone neoplasm [*] Suspected osteosarcoma case 1 herein included
Lung	Petrini et al. [26]; suspected lung neoplasm Guimarães et al. [27]; presumptive pulmonary neoplasm Gazzoni et al. [28]; presumptive lung primary neoplasms Stieglitz et al. [29]; suspected Ewing sarcoma metastasis to lung	Lung	Shubitz and Dial [16]; suspected metastatic neoplasms
Gonads	Halsey et al. [30]; presumptive testicle neoplasm Ellis et al. [31]; suspected ovary malignancy	Gonads	Ramírez-Romero et al. [14]; suspected primary neoplasm in testicle
Brain	Komotar and Clatterbuck [32]; plaque meningioma	Brain	Bentley et al. [33]; suspected neoplastic masses
Skin subcutis	Schwartz and Lamberts [34]; suspected squamous cell carcinoma Crum [35]; suspected mycosis fungoides Hirschmann [36]; since the original descriptions mycosis fungoides was suspected	Skin subcutis	[*] Herein, associated to ribs; suspected neurofibroma, case 3
Abdomen	Eyer, et al. [37]; suspected peritoneal malignancy		

In reviewing the literature, it was surprising to find only two reports in dogs in which coccidioidomycosis was masquerading malignancies. One case was from a dog erroneously diagnosed by the attending clinician with a testicular tumor [14], and another two dogs with a suspected heart-base tumor [17]. Systemic mycosis including coccidioidomycosis should be at top differential diagnosis in dogs with chronic debilitating disease, particularly if there is evidence of generalized lymphadenopathy, lameness, skin nodules or ulcers that do not heal. Another crucial factor is if the dog has been living or has travelled to zones where coccidioidomycosis is known to be endemic [16, 38]. It is noteworthy to mention that neoplasm and coccidioidomycosis can simultaneously occur in the same dog. A good example of this type of comorbidity was reported in a mature dog with history of lethargy, inappetence and generalized lymphadenopathy diagnosed afterward with disseminated coccidioidomycosis and multisystemic lymphoma [20]. Interestingly, the clinical signs and lymph node enlargement in this dog improved notably after antifungal treatment and chemotherapy [20].

Human coccidioidomycosis is highly prevalent in the northern Mexico particularly in regions neighboring the USA. According to several epidemiological surveys conducted between 1994 and 2005, the prevalence of *Coccidioides* spp. infection recognized by intradermal reaction, ranged geographically from 9.2 % in Tijuana, Baja California to 93 % in Matamoros, Coahuila [8]. The overall rate calculated for Mexico is 1.6 % [8]. As it may be expected, the prevalence rates in northern Mexico are comparable to those reported for humans in the southern USA [6, 7].

It is worth noting that the three dogs reported in this study were from Nuevo Leon, the Mexican state with the highest prevalence of human coccidioidomycosis in the country. A large study conducted between 1983 and 2000 in 4598 autopsies in Nuevo Leon showed that 31 (0.67 %) of the cadavers had evidence of coccidioidomycosis [39]. Although the studies are different, the higher rate of cases in human autopsies is only the double than the positive biopsies encountered here in much less samples. Since the mode of infection and lesions are similar in dogs and humans, the dog has been considered as sentinel as well as animal model for this disease [40–42].

The granulomatous lesions incited by *Coccidioides* spp. in dog are dominated by T lymphocytes [43]. However, the inflammatory reaction is not specific and comparable lesions can also be caused by other fungal and algal infections that share some histopathological images with coccidioidomycosis, such as paracoccidioidomycosis [44], blastomycosis [45], protothecosis [46] and chlorellosis [47], that naturally occur in the dog. Nonetheless for all of these infections, if tissue is available, histopathology is the elected procedure [2]. On the other hand, molecular procedures would probably be the upcoming procedures, but nowadays histopathology is the most accurate diagnostic tool. In the present study, the difficulties to work with paraffin-embedded tissues were solved not easily and this has been mentioned recently [4]. However, the positive result in the tissue from case 3 correspondent with *Coccidioides posadasii* is very interesting and is in accordance with previous studies which report this species as more prevalent in the Northeast in Mexico and the corresponding Southeast of USA [6, 7]. Future studies are required to characterize the species involved in natural cases of coccidioidomycosis in dogs in Nuevo Leon, Mexico.

Despite of the few cases encountered here, coccidioidomycosis has to be considered into differential diagnosis of proliferative lesions suspected of neoplasm, particularly in Nuevo Leon, state included in the endemic zone of Mexico.
